# Health-related dimensions of fishers for sustainable commercial fisheries in the Atlantic Gulf of Guinea: Ecological and social assessments

**DOI:** 10.1016/j.onehlt.2025.100972

**Published:** 2025-01-10

**Authors:** Isa Oalekan Elegbede, Saud. M Al Jufaili, Toheeb Lekan Jolaosho, Babalola Tesleem, Awe Folalu Adekunle, Olarinmoye Oluwatosin Modupe, Salisu Monsuru Adekunle, Adedeji-Adenola Halimat

**Affiliations:** a Department of Fisheries, Lagos State University, Nigeria; b Department of Environmental Planning, Brandenburg University of Technology, Germany; c Department of Marine Science and Fisheries, Sultan Qaboos University, Oman; d Spatial Science, Islands and Sustainability, University of Groningen, Netherlands; e Stony Brook University, NJ, United States of America; f Department of Agricultural Science, Faculty of Technical and Vocational, Universiti Pendidikan Sultan Idris, 35900 Tanjung Malim, Perak, Malaysia; g University of KwaZulu-Natal, Westville Campus, Durban, South Africa; h Sustainable Management of Fishries Resources, University of Las Palmas de Gran Canaria, Spain; i TealNex LLC, United State of America

**Keywords:** Socio-ecological systems, Health conditions, Industrial shrimp fisheries, Safety measures, Sustainability, Fishing fleets, Certification programmes, Sustainable development goals

## Abstract

Health considerations are seldom integrated into non-state voluntary sustainability certification standards, despite industrial fishing being one of the most hazardous occupations, often resulting in significant health risks for fishers. These challenges not only affect individual fishers but also have broader implications for the socio-ecological sustainability of fisheries. This study explores the effects of certification programs on industrial fishing activities and their health and safety dimensions within a socio-ecological framework, focusing on the Atlantic shrimp fishery in Nigeria. A mixed-methods approach was employed, combining qualitative and quantitative data collection techniques. Data were gathered from experienced employees of Friend of the Sea (FOS) certified and non-certified (Business-as-Usual, BAU) industrial fisheries through questionnaires, complemented by semi-structured interviews with selected key informants. The study examines the relationships between demographic variables, health-related indicators, and certification program participation using multivariate analyses, including Chi-square and standard logistic regression models. The findings revealed that fishers participating in the FOS certification program reported better health conditions, lower risks, and higher insurance coverage levels than their BAU counterparts. Certified fishers had greater access to sick leave and annual days off and were more likely to be physically fit for work. Conversely, fishers in the BAU group faced significantly higher health risks and casualty rates during fishing operations. These results underscore the critical role of health-focused certification programs, such as FOS, in improving the well-being of fishers, thereby enhancing the socio-ecological sustainability of Nigeria's shrimp fishing industry. The study highlights the importance of integrating health considerations into sustainability certification standards. By prioritizing fisher's safety and well-being, such programs contribute to the sustainable management of fisheries resources and environmental protection, aligning with the principles of the One Health framework.

## 1 Introduction

Many commercial fishers often overlook the importance of health, despite its direct impact on the effectiveness of fishing efforts and overall productivity [1,2]. Commercial-scale seafood fishing in ocean and coastal environments is a risky activity with adverse health effects [3,4]. Unfavorable working conditions, unsustainable maritime operations which include overboard or port drowning, exposure to toxicants, incidental entanglement with machineries, crush injuries [5], noise, and vibration, and external factors such as weather are responsible for health risks and in some cases, the deaths of workers [6,7]. Larger vessels always have onboard emergency medical personnel, but there are limitations to the health problems they can solve. Onboard medical personnel are often well-equipped enough to address minor issues such as cuts, bruises, seasickness, skin infections, headaches, or muscle aches. However, they are limited in their ability to address complex health issues such as crush injuries, fractures, amputations, head injuries caused by machinery entanglement, chemical exposure which requires specialized medical expertise, heart attacks, or other cardiovascular conditions that require immediate advanced care [8]. The inadequacy in managing severe health issues demonstrates the need for improved medical resources or alternative strategies in maritime operations to better protect worker's welfare. Conversely, small-scale fishing is considered to be a solitary activity that might lack the ability to request emergency support in the case of illness or injury [9]. In many underdeveloped nations, fishing companies often do not have experienced medical practitioners on board. However, to ensure successful and efficient fishing operations, it is essential that fishers are in good health, certified as medically fit before starting their activities, provided with comprehensive health insurance, and have access to ready-to-use shore-based medical facilities [10].

The socio-ecological dimensions and sustainability of fisheries demand appropriate consideration of some major elements such as environmental, occupational, and social factors, which are critical indicators for assessing the safety and well-being of fishers [11]. Meanwhile, this aspect (human health consideration) in relation to fishing operations has frequently been neglected. Fisheries governance systems have often struggled to integrate social and economic considerations of health and well-being, into policy frameworks [12]. Notably, optimal safe working conditions and good health enhances the capacity of fishers to support sustainable fisheries. However, industrial fishers frequently encounter adverse health conditions and are subjected to numerous occupational hazards, particularly during high-fishing operations on the high Sea [13]. Unsustainable fishing practices, including overfishing, destructive fishing practices, water pollution and habitat degradation threatens the yield and abundance of fisheries resources [14]. This compels fishers to operate further offshore, thereby increasing their exposure to severe weather, strong currents, and prolonged physical labor. In some cases where diminishing fish stocks and yields are evident due to environmental degradation, fishers are forced to adopt more labor-intensive practices to sustain their livelihoods [15]. This poor working conditions, coupled with inadequate safety guidelines, limited access to protective equipment, and exposure to toxicants pose adverse health risks to fishers. Furthermore, restricted access to healthcare services in coastal communities frequently results in fishers lacking prompt medical attention for injuries, illnesses, or mental health concerns stemming from the physically and emotionally demanding nature of their occupation [16]. These challenges not only jeopardize the safety and well-being of fishers but also impede fisheries sustainability, resulting in a vicious cycle whereby unsustainable practices harm human health and ecological systems.

Commercial fisheries are crucial for groups of fishers from ecological, socioeconomic, and economic perspectives. The interactions between socioeconomic factors (working conditions, access to healthcare, etc.) and ecological factors (environmental conditions, natural risks, etc.) are essential for understanding the health status of fishers. The risk and impact of hazards faced in fishing operations are directly related to these interactions [[17], [18], [19], [20], [21], [22]]. Understanding the potential risks and consequences of health hazards encountered during fishing operations is important for the sustainability of aquatic ecosystems and their essential resources. This dimension aligns with the aim of promoting one health, given it emphasizes on the interconnection of human, animal, and environmental health. Notably, it links SDGs 3 and 14 and emphasizes the importance of achieving sustainable development aims. A crucial aspect of SDG 3 focuses on ensuring worker health and safety. As a sign of 3.8.1 (encompassing health services) and 3.8.2, which focus on household health spending, Target 3.8 focuses on the realization of universal healthcare services [23]. The assumption is that the fishing industry would oversee and protect worker health and safety through the provision of on-call medical personnel to aid fishers in emergencies and adequate insurance coverage for occupational accidents [24]. These responsibilities have been further emphasized by several sustainability certification programs that recognize health and safety as essential measures for sustainable fishing practices [[25], [26], [27], [28]].

The Friend of the Sea (FOS), guided by its commitment to social responsibility, emphasizes improving fisheries as a way to uphold the social values of fishers, focusing on reducing risks and preventing injuries and fatalities. People prioritize health and safety procedures in Asia when choosing a certified fish product [25]. The economic expansion of fishing operations, including worker productivity, is supported by safe working practices. However, evidence from a research study has shown that fishing companies are gradually compromising the welfare of their workers by reducing investments in essential health and safety protocols to minimize expenses. Chen et al. [29] found that cost-reduction strategies in the Chinese fishing industry resulted in insufficient investment in safety equipment, including personal flotation devices and protective apparel, leading to a rise in workplace injuries and fatalities. Similar trends have been noted in the Norwegian maritime sector, where insufficient investment in safety technology and equipment maintenance has increased risks for workers, including entanglement with machinery and exposure to hazards [5]. Hence, the International Maritime Organization (IMO), the International Labour Organization (ILO), and fishing firms must all work together to ensure that employee health and safety are prioritized [30]. Potts and Haward [31] claim that the growing interest of fishing industries in ecolabel certification results from the heightened concerns about health and safety. This demonstrates fishing industries are beginning to recognize the necessity for sustainable practices that not only protect the environment but also ensure the welfare and safety of fishers.

The commitment of a company to its employees also affects their health and safety, as evidence in the quality of working conditions and support provided. Respected workers who are held in high esteem for their dedication and work ethic cautiously carry out their responsibilities to ensure their safe return home to their families [32]. This shared respect and understanding cultivates a harmonious relationship that mutually benefits all the parties involved. Due diligence in terms of health and safety is an investment rather than a cost. Despite the significant dangers associated with industrial fishing operations, many fishing companies continue to neglect essential safety regulations [33]. As productive employment relates to working decently in fisheries, the health and safety problems inherent in SDG 14 highlight the notion of decent employment possibilities in the fisheries value chain [34,35]. Many fishers experience physical discomfort, exhaustion, infections, hearing issues, and stress, which affect their everyday lives [2,36]. Similarly, workers in various occupations face adverse health conditions, which are categorized based on the provision of the International Classification of Diseases (ICD-10) [37]. These conditions include mental and behavioral disorders, such as psychological discomfort and depression (F30–F39); neoplasms, including cancer (C00–D49); endocrine, nutritional, and metabolic diseases like diabetes mellitus (E08–E13), which is often associated with elevated blood sugar levels; and circulatory system disorders such as hypertension (I10–I15).

Health and safety considerations are significant factors in ensuring the well-being of fishers and play a vital role in enhancing the overall efficiency and productivity of the fishing sector [2,26]. Similarly, the health of fishers plays a critical role in advancing sustainability goals under certification standards like FOS [38]. Competent and healthy personnel are crucial for effectively overseeing and implementing sustainable practices, including proper monitoring of fish populations, and bycatch mitigation to ensure ecological equilibrium [39]. Nevertheless, adverse health outcomes due to unfavorable working conditions, inadequate access to healthcare, and exposure to environmental toxins can impede the effective implementation of such programs, as ailing or injured fishers may be less capable of adhering to sustainability standards or participating in conservation efforts. Additionally, socioeconomic disparities within fishing sector, including financial insecurity and competition for limited resources can exacerbate the challenges of implementing sustainable practices [40]. Researchers have documented the ecological effects of capture fisheries activities and trades in the Atlantic Ocean [41], as well as the health risks faced by coastal artisanal fishers [42] and small-scale fish processors [43] in the Nigerian context. However, the safety and health risks of industrial fisheries, and also the efforts of sustainability certifications on Nigerian fishers, particularly those operating in the Atlantic, have not yet been thoroughly investigated by researchers. This highlights a clear and significant gap that needs to be addressed, which further emphasizes the importance and relevance of this research. Anecdotal evidence demonstrates that fishers have worse health conditions and are less likely to access healthcare than other occupational groups due to intricate organizational policies and the pervasive social norms that demoralize help seeking among this group [2]. To this end, this study explores the extent to which organizational sustainability in the fisheries sector influences the health of fishers in the commercial fishing industry. The main objective is to investigate how specific aspects related to fisher's health may affect the effectiveness of sustainability efforts of the Friend of the Sea (FOS) program. This study further examines the efficacies of the FOS certification intervention in comparison with the business as usual (BAU) fishing operations. This study intends to establish a distinct correlation with the one health framework by analyzing health-related aspects within the socio-ecological context of fisheries, which acknowledges the interconnected health impacts on fishers, the sustainability of fisheries, and the surrounding environment.

## 2 Methodology

### 2.1 Study area

#### 2.1.1 Geographical and ecological features of the study location

**Fig. 1:**
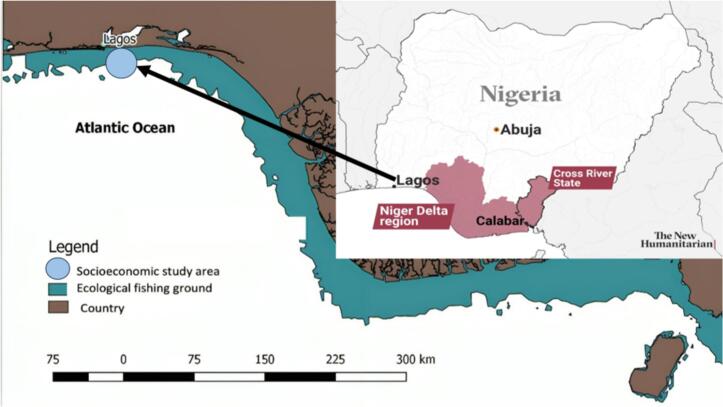
Map of Nigeria showing the Atlantic Ocean, Gulf of Guinea.

Nigeria is located between latitudes 4° 16′ and 13° 52′ N and longitudes 2° 49′ and 14° 37′ E. The nation is estimated to be 923,768 km2 in size, with 13,000 km2 of inland water and 910,768 km2 of land. Its coastline is divided into geomorphological sections and spans 853 km [44,45]. Nigeria borders the Benin Republic to the west, Niger to the north, the Atlantic Ocean and the Gulf of Guinea to the south, and Cameroon and Chad to the east (Fig. 1). It is estimated to be 1200 km long from east to west and 1050 km long from north to south. It is made up of lowlands, valleys, plateaus, and mountains. Its ecological features include dry savanna and tropical forests, both of which are rich in flora and fauna [46].

There are both saltwater and freshwater swamp forests along the coast. Around the lagoon and along the coast, the saltwater stretches for roughly two kilometers [47]. The tropical lowland rainforest is characterized by the presence of small trees, herbs, and shrubs, while the mangrove regions also include freshwater forests [48]. Mangroves along the lagoon are part of the mangrove swamp forest, which also includes the deltaic swamp forest, which includes Raffia palm and mahogany, in the flood plains of the Niger Delta. Around Ibadan, Benin, Niger Kondo, and the Cross River, as well as in the area bordering Cameroon, are areas of inland rainforest [49].

Nigeria experiences both dry and wet seasons, with temperatures ranging from 22 °C to 36 °C [50,51]. With 330 cm of annual rainfall, the southern region is more affected by the wet season, while the southern region is more severely affected by the dry season due to high temperatures and aridity [50]. Harmattan, a dusty, low-temperature wind from the Arabian Peninsula of the Sahara, is what defines the dry season. The wet season begins in April and ends in October, while the dry season begins in November and lasts until March [50,51]. Wet tropical zones near the coast, semiarid tropical zones in the north, and seasonal wet and dry tropical in the center are the three climatic zones that make up the nation [51]. The wet tropical region in the south is characterized by consistently hot and humid conditions, with balanced annual temperature and humidity levels. Daytime and nighttime temperatures average 35 °C and 21 °C, respectively, and annual precipitation reaches approximately 3000 mm. In contrast, the northern Sahel region features a semiarid tropical climate, receiving about 250 mm to 500 mm of annual rainfall [52]. The sea experiences seasonal wet and dry conditions due to temperature fluctuations between 46 °C and 6 °C. During the wet season, rainfall typically ranges from 760 to 1500 mm.

Nigeria's shoreline is carved out by mangrove ecosystems in the lagoon to the southwest and by the Niger Delta to the east, which connects the Benin, Escravos, Forcados, Ramos, Dodo, Middleton, Fish Town, Nun, Brass, San Bartholomeo, Bonny, and Opobo Rivers and Estuaries [53]. The majority of Nigeria's fish resources are found in the Niger Delta, which is located in the east and is the second-largest delta in the world at 500 km [44]. Twelve nautical miles (NM) and two hundred (NM), or 22,224 km and 370.40 km, respectively, make up the territorial zone and exclusive economic region [54]. Correspondingly, Nigeria's whole continental shelf area is 41,000 km2. According to Zabbey et al. [44], it is composed of inland waters from Benue, the northeast and northwest of the Niger River, and the Lokoja confluence. These waters then flow south-south and into the southwest and south waters, where they empty into the Atlantic Ocean. The most valuable fish resources can be found in the thermocline between 30 and 60 m below the surface due to the hydrographic features of the Nigerian coast [55], which favor fish production around the shelf with water temperatures above 25 °C at 50 m depth. At the surface, the salinity along the coast is 34%, while at depths greater than 400 m, the oxygen content significantly decreases [56].

The topography of the Nigerian coastline includes mud and sand deposits that affect fish populations in the wild and make fishing easier. Due to river flow, the Niger Delta area to the east of the coast also contains sand and mud. The clays are located at a shallow level along the central lagoon of Ondo, heading west, and are buried in the delta [44]. The length of the continental shelf varies along the coast, reaching 35 km along the Lagos axis, 64 km along the Cape Forces, and 75 km along Calabar in the Delta region. From Calabar westwards to the Avon and Mahin canyons, there are more canyons along the coastline. The Nigerian coast is divided into four main sections: the Niger Delta, the central transgressive mud coast, the barrier-lagoon complex, and the strand coast that stretches from east to west [57].

#### 2.1.2 The socioeconomic attributes of the study area

Nigeria is considered the most populous African nation, with an estimated 190 million citizens [58]. With a 2.4 % annual population growth rate, migration, fertility, and mortality are important factors influencing population growth. By 2050, 392 million people are predicted to live in Nigeria, making it the country with the fourth largest population in the world [59]. The nation is also recognized as a low-income economy, and its rapid population growth has raised concerns, highlighting the need for the sustainable development of its socioeconomic system [60].

The disparity between rural and urban areas contributes to another socioeconomic opportunity gap, as a greater proportion of the Nigerian population (roughly 52.1 % of the total area), lives in impoverished rural areas. In Nigeria, social values and beliefs are influenced by religion, and the diverse social values and beliefs that are widely held across the nation have an impact on a number of poverty-related variables, including age, employment, household size, gender, marriage rate, and age at birth [61]. In addition, there are 37 births per 1000 people in Nigeria. Approximately 47 % of the people in Nigeria lives in urban region. The largest metropolitan areas are Lagos, home to 13 million people; Kano, home to almost 4 million people; Ibadan, home to 3 million people; Abuja, home to about 2 million people; and Port Harcourt, home to 2 million people. According to Anyanwu [62], decreased productivity as a result of lost jobs or lower income contributes to an increase in poverty as a social indicator, particularly as individuals grow older.

Social values in Nigeria are directly correlated with marital status. A total of 69.8 % of married couples live above the poverty line, which is the average percentage of married people. By comparison, only 54.74 % of divorced individuals remain above the poverty line, despite 61.89 % of them being in this situation. According to Anyanwu [62], monogamous families in Nigeria have the highest social standards. In Nigeria, the size of a household, which includes parents, children, and extended family members, is influenced by marriage. However, social values decline with household size; the average household size with one person has the lowest rate of poverty (22.6 %), while the rate of poverty in a household with seven or more people is higher (97.61 %) [62]. Low literacy rates are primarily responsible for the escalating poverty in Nigeria, particularly in the northern regions of the country. The low level of development in Nigeria is caused by the approximately 38 % illiteracy rate of its population. Socioeconomic underdevelopment is also significantly influenced by corruption, which increases the poverty rate by causing an unequal distribution of wealth among the populace. Weak political institutions and national instability are the main causes of this predicament [63].

The social dynamics of this study incorporate the economic perspectives of the Nigerian context. With an estimated $1 trillion in GDP as of 2015, Nigeria has become the largest economy in sub-Saharan Africa. The nation's economic base is the middle-class, and its 2016 unemployment rate was 13.9 % [64]. Employees in the public sector, nongovernmental organizations, and other career-oriented positions, typically experience above-average financial success, significantly above the national poverty line [62]. On the other hand, the jobs with the greatest rates of poverty among working Nigerians are local cooperative jobs and self-employed farming and fishing. However, industrial fisheries are one of the main sectors with the highest income generation in Nigeria. The fisheries sector, including both aquaculture and capture fisheries, contributes approximately 5.4 % of the Nigeria's GDP [65]. Data from the Fisheries Committee for the West Central Gulf of Guinea on vessel licensing indicates that annually, between 200 and 300 vessels along the Nigerian coast have been authorized over the past decade to participate in fishing and shrimping activities, collectively yielding between 23,000 and 34,000 tons of seafood each year [66]. In addition, the number of trawlers licensed has been steadily increasing, with about 200 trawlers currently registered annually. Nigeria hosts approximately 40 trawling companies, many of which are members of the Nigerian Trawler Owners' Association (NITOA). The majority of these companies manage small fleets, usually comprising fewer than four vessels, and are primarily owned by Nigerians. Larger companies, operating fleets of four or more vessels, are often joint ventures involving foreign investors. Vessel licensing is regulated under the provisions of Nigeria's Fisheries Law and Regulations. Currently, about 36 fishing companies own approximately 271 Nigerian-flagged vessels licensed to shrimp within the country's territorial waters. Approximately 40 % of West Africa's population inhabits coastal regions within the larger Gulf of Guinea, where fisheries are essential for sustenance and economic support. In Nigeria, the fisheries sector employs approximately 790,000 fishers, whereas the coastal region of West Africa supports around 9 million individuals in fisheries-related occupations [34]. Although more than half of fishers participate in inland fisheries, marine artisanal fisheries account for the highest proportion of processors at 42 %, followed by 30 % in inland fisheries and 28 % in industrial fisheries.

### 2.2 Research context

The study design adopted a mixed method research approach to examine the intricate socioeconomic andecological systems of the Nigerian shrimp industrial fishery. This approach was informed by the research aim, guiding the adoption of appropriate data collection strategies and methods [67,68]. The primary focus of this comparative assessment is to examine the effectiveness of the FOS sustainability certification program using health-related indicators of fishers. It is hypothesized that there would be no significant difference in health and safety outcomes between FOS-certified fishers and BAU fishers, regardless of the influence of sustainability certification standards. This study considers the health-related and socioeconomic variables in the shrimp fishery for the FOS and BAU groups in the context of the socioeconomic and ecological frameworks. The absence of clear boundaries between the fishing areas of the companies distinguishes this scenario from terrestrial ecosystems. Both certified and non-certified fishers operate in the same regions, target similar species and adhere to guidelines from national, regional, and international bodies such as the FAO. In Nigeria, the Federal Department of Fisheries (FDF) issues certifications that enable fish and seafood products to meet international standards, granting access to global markets such as Europe, the USA, and Asia. However, challenges such as inadequate infrastructure, compliance with stringent international regulations, and ensuring sustainability in fisheries management persist, hindering the full potential of the sector. The introduction of a third-party certification program is expected to promote sustainable market practices and induce sectoral changes. The presence of this certification program, coupled with available data, allows for comparison with fisheries employing conventional practices. Certification relies on indicators with distinct criteria, that are subject to third-party accreditation.

Demographics are a range of social measurements that may represent the conditions or experiences of a particular social group or community [69]. Therefore, demographic information may be considered an inventory of the processes and collectiveness of the human population and their potential in influencing their environment. This study considers age, gender, and marital status as relevant demographic variables due to their relative and active proportionality to income according to the United Nations Department of Economic and Social Affairs index [70]. The selection was driven by previous studies which found that demographic variables such as age and gender are known factors that impact productivity [71,72]. Similarly, it has been reported that the participation level in fishing activities is affected by demographic parameters such as age and gender [73,74]. For instance, younger fishers may exhibit greater physical activity and proficiency in undertaking strenuous fishing tasks, perhaps resulting in increased yields and income. Conversely, older fishers may rely on experience and established networks, which can positively impact their earnings. Gender also plays a significant role in household income, as industrial fishing operations involve physically intensive and risky tasks. Marital status of fishers is also important, as this could affect workforce involvement. For instance, unmarried fisher might be more willing to spend extended periods at sea, taking on longer voyages that could result in higher income. Similarly, married fishers may have greater responsibilities such as supporting their families, which can drive them to work harder, take longer trips, or seek higher-paying opportunities.

### 2.3 Research methods, sample selection and sampling techniques

**Fig. 2:**
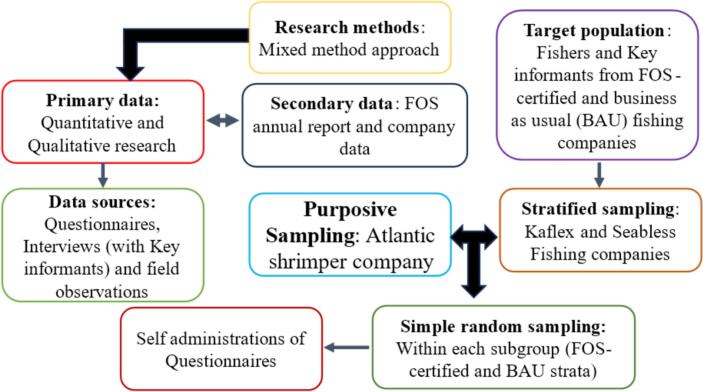
Research methods and sampling techniques of the study.

Fig. 2 depicts the flowchart that delineates the research design, sampling techniques and the data gathering processes of the study. This study does not have the resources to research the entire fisher population. Therefore, sampling a representative portion of the population was considered appropriate. Based on the population targeted in the study, mixed sampling techniques were adopted. There are guidelines for selecting a mixed sampling technique based on the logic and conformity of the research questions and hypotheses. Equally, assumptions of the random and purposive sampling techniques were considered. The study collected both qualitative and quantitative data, mainly from primary sources, while taking into account the ethics, feasibility, and efficiency of the sampling approach [75]. The quantitative data was collected through questionnaires, while the qualitative data was obtained via semi-structured interviews and visual observation. Additionally, we collected secondary data from the FOS annual reports regarding the fishing and health-related services provided to the FOS-certified company.

The fishing companies and the participants were recruited using purposive sampling as well as simple random and stratified sampling respectively, due to the structure of the research design, the aim of the research and the nature of data used. The combination of these sampling approaches was used to adhere to the comparative framework of this research [76]. One of the reasons for examining the sample size is to analyze the kind of fisheries management practised by both groups. The study participants were mainly fishers from one FOS-certified entity, [Atlantic Shrimpers Limited (ASL)], and two business-as-usual (BAU) companies, (Seabless and Kaflex). The selected industrial fishing companies are mainly located in Apapa area of Lagos State in Nigeria but operates majorly in the in the Atlantic Gulf of Guinea.

ASL fishing industry was chosen as a representative of a FOS-certified company because of its alignment with the sustainability practices promoted by FOS in Nigerian shrimp fisheries. Moreover, the group is the sole entity certified by sustainability standard in the Nigerian fisheries sector. ASL possesses the largest fishing fleets in Nigeria and West Africa, having over 70 fishing trawlers. The average overall length (LOA) and gross registered tonnage (GRT) of the ASL are 23.2 m and 134.6 metric tons, respectively. This company has been active in shrimp trawling since the 1990s but got certified by the FOS in 2015, which provides a benchmark for evaluating the impact of sustainability certification on operational and labor conditions [77]. This enterprise has engaged in shrimp trawling since the 1990s and received certification from the FOS in 2015, which enables entities to establish a standard for assessing the effects of sustainability certification on their operational and labor conditions [77]. ASL has recently made investments in a number of fisheries and health sustainability tools, including sound traceability systems, net retrieval, detection systems, as well as health and safety equipment such as protective gear, and overboard detection systems. ASL stands out as the top-certified FOS company because of its dedication to the health and safety of its employees, which aligns with the FOS certification requirements.

The BAU companies, Kaflex and Seabless, were chosen since they are still the least likely to obtain regulatory sustainability certificates. Seabless, for example, only has five fishing boats, each with an average length of attack (LOA) of 22.03 m and a gross tonnage (GRT) of 123.5 metric tons [78]. The company was founded as a domestic Nigerian enterprise that focuses on commercial and industrial fishing in Nigeria and West Africa, including the processing and packaging of seafood products for both local and foreign markets. A Nigerian navy vessel was recently reported to have saved 12 crew members from a capsized fishing vessel owned by the Seabless fishing company [79]. Conversely, Karflex, which was founded in 2003, places less emphasis on sustainability certifications that guarantee the welfare, good health, and safety of fishermen and more on business ventures such fish packaging, sorting, product storage, and exporting. The company owns twelve standard vessels, each with an average size of 21.65 m and a fishing capacity of 1118.75 metric tons. These values are lower than those of ASL, even though they fall within the typical vessel LOA range of 23.00 m to 26.30 m and the GRTs of 120.43 to 148.8 for Sea Fisheries (Licensing) [80].

After the selections of the companies, a simple sampling technique was used to collect data randomly from fishers within the FOS group. On the other hand, a stratified probability sampling technique was adopted for the BAU group. Furthermore, this group partitions into strata groups of industrial fishing companies such as Kaflex and SeaBless. Subsequently, simple random sampling was conducted in each of the BAU subgroups to retrieve data with self-administered questionnaires, which were used as control groups to evaluate the impacts of FOS in the fisheries.

### 2.4 Research sample size

The determination of sample size is essential to achieving the aim and objectives of the study. Thus, comprehensive research should rely on effect size, the significance factor, and the power of statistics to ensure the validity of the effect. The sample size is the minimum required group size to verify that the difference between the two populations is significant, that is, proportionate to the number of confidence levels and power [81]. Our case study involves two independent groups, the FOS and the BAU. The estimation of the sample size for the two groups follows the assumptions by recognizing valid differences between the groups, which provides an indication of power. The power is always accepted to be between 80 % and 100 %. The significance level or *p*-value is the limit by which the null hypothesis is rejected, (5 %). The assumed proportion (p) for both groups whose base can be considered based on existing literature or research in which the differences are examined (p1-p2) (Eq. (1)). Thus, the sample size was estimated with the following formula for two independent proportions for two groups [81].

(1) $$n=\frac{\;{\left(Z_{\alpha /2}+Z_{\beta }\right)}^{2}\;\left(p1\left(1-p2\right)\right)+p2\left(1-p2\right))\;}{{\left(p1-p2\right)}^{2}}$$

where Z_α/2_ = the critical value of the Normal distribution when α is 0.05 with a 1.96 and 95 % critical value and confidence interval respectively. Z_β_ = the critical value of the normal distribution when β is 0.2 with 0.84 and 80 % power. $P_{1}$and $P_{2}$ are the sample proportions of both groups.

Based on the assumption that the data are normally distributed, the sample size was determined, via a two-way hypothesis. The population of the fishing community is 500,000 made up of individuals involved in the industrial fisheries. Based on the consideration and input used, this study estimated a sample size of 250 for each of the groups making a total of 500 individuals. The questionnaires were not administered simultaneously due to cost and bureaucratic obstacles. However, these uncertainties affect the respondent's feedback, given that only 198 and 218 questionnaires were returned with percentages of 79.2 % and 87.2 % for FOS and BAU groups respectively, making a total of 416 retrieved of the total 500 questionnaires.

### 2.5 Sources and methods of data collection

For desktop research, the Brandenburg University of Technology (BTU) library was highly engaged, using various library tools and materials such as online documents, reports, theses, books, journals, articles, and blogs. In particular, FOS audit reports have been instrumental to the progress of this research. The published FOS audit reports for 2011 and 2015 were sourced to extract secondary information regarding the services provided, requirements, and criteria that need to be met before a fishing company could be certified. We also considered direct observations and the adoption of questionnaires to obtain data regarding demographic, socioeconomic, and health information from the selected fishers. Lastly, the study considered the use of key informants for open-ended, semi-structured interviews. Direct observations were recorded at the fishing operation sites and landings, including details of the catch, type of effort, vessel, and gear specifications, including horsepower, fuel usage, energy usage, etc., and discussions related to the scope of the study were also held with experienced workers. This observational activity enhances the smooth administration of interviews and questionnaires [82]. The questionnaires were distributed to the fishers and collected within one week. This approach ensured that the surveys were completed accurately and efficiently while minimizing errors or data inconsistencies. Their inputs were intended to complement other aspects of data collection, such as field observation and semi-structured interviews [83]. The research interview was a data collection format that used standardized questions and responses to ensure consistency, intending to validate data from other sources [83].

Interviews with key informants provided invaluable qualitative information regarding health insurance coverage for fishers, annual leave of crew members, challenges encountered at sea, and injury/accidents during operations to complement other research methods (see supplementary file). The key informants interviewed were from the FDF in Nigeria, industrial fishing companies (IFC), academic and research institutions (ARI), and the NITOA, which are all considered major stakeholders in the fishing industries. These informants were selected based on their expertise and represented key stakeholders in the fisheries industry. The open ended semi-structured interviews allowed for detailed responses, thereby enhancing the depth of our research framework. Some interviews were conducted in offices, while others were conducted over the phone. Overall, the data collected include demographic data, such as age, gender, marital status, and health status, and safety-related variables such as type of health issue, confirmation of health fitness before work, type of health issues from work, accessibility to sick and annual leave, and inability to work due to health issues. Despite challenges such as non-disclosure agreements, interviews were conducted with a focus on triangulating data collected from other sources.

### 2.6 Triangulation, reliability and validity

This research necessitates the use of multiple research methods, to define the sources of data, analyze and validate the findings and make a high-quality assessment. When multiple structures are considered for research, bias from theories, data, or methods can be omitted, thereby increasing the reliability of the results [84]. The methodology for this study involved collecting data through field observations, document reviews, and questionnaires, utilizing sampling strategies aligned with the research scope and objectives. However, triangulation as a method has its limitations, such as challenges in replicating both quantitative and qualitative data and the significant resources required to implement it effectively [84]. However, this approach has contributed to the quality of this research, by enhancing the reliability and validity of the findings while tackling errors emanating from the research methods and improving the accuracy of the measurement tools [84,85]. The validity of this research was ensured by probing the primary data collected with the secondary data. Field observations were used to validate some of the information retrieved from the desktop studies. The interview questions and questionnaires followed a consistent and logical format, thereby indicating a reliable outcome [86].

### 2.7 Data analysis and multivariate techniques

The collected demographic, socioeconomic and organizational datasets subjected to analysis. The dataset was cleaned, and assumptions (sampling, independence, normality, homogeneity of variance and linearity) were checked with validations to bridge the gaps between uncertainties and understanding [87]. The normality of the data was done using the Kolmogorov-Smirnov and Shapiro-Wilk tests. The descriptive graphical analysis produces radar plots and tables to visualize the quantitative data with a logical comparison between the FOS-certified and BAU groups before and after certification, allowing adequate interpretations of the variables [[88], [89], [90]]. The obtained quantitative datasets were imported into Microsoft excel sheets and exported to IBM-SPSS Version 26.0 for statistical analysis. The recorded audios (qualitative data) obtained during the interviews were transcribed into texts with TurboScribe. In addition, chi-square test was used to analyze the significant differences and relationships between the FOS-certified and BAU groups. Logistic regression was adopted to compare the log odds of the grouped indicators. Below are the detailed descriptions of these models and analyses.

This research presents the logistic or logit regression model to examine the log odds scalable relationships between the independent and the dependent variables in categorical data [91]. Logistic regression was developed to identify factors associated with the FOS certification programme based on 95 % confidence intervals. The variables were determined with the options of the FOS and the BAU groups as the binary dependent variables known as a response. Moreover, the socioeconomic parameters are recognized as independent variables (predictors). This study treats the BAU group as a dependent variable representing the baseline category. The first category of the independent variables was designated as the baseline group for comparison with other groups. Consequently, the necessary interpretations were provided to analyze the findings [91,92]. The overall baseline model for logistic regression is as follows:

(2) $${\mathrm{yi}}^{*}=\beta_{0+}\;x_{i}\;\beta +e_{i}$$

Given that the dependent variable is categorized, it is necessary to use.

(3) $$C_{x}\;\left(x\right)=\text{In}\;\left(\frac{P\;\left(Y\leq j|x\right)}{P\;\left(Y>j|x\right)}\right)$$

(4) $$\text{In}\;\left(\frac{⅀\mathrm{pr}\;\left(\mathrm{event}\right)}{1-⅀\mathrm{pr}\;(\mathrm{event}}\right)=\beta_{0}+\beta_{1}X_{1}+\beta_{2}X_{2}+\ldots +\beta_{k}X_{k}$$

where yi* is the ordinal outcome (a latent variable) describing the likelihood of FOS certification. β_0_ is a constant and intercept, β is a regression coefficient known as a vector of parameters, “xi” is the collection of independent explanatory factors or variables, and “e_i_” is the error term with standard logistic distribution and independent variable x_i_.

The confidence level for this test is 95 % with a significance level of 5 %. The demographic data were incorporated into the model as codes of the independent variables FOS as one and BAU as zero. Data obtained from the research were analyzed for the logit model using STATA software v.17.0.

### 2.8 Ethical consideration and approval

Prior to conducting the research, ethical approval was obtained from the Ethical Review Board of Brandenburg University of Technology, Germany, as well as from the participating industrial fisheries organizations. The approval process required the submission of a comprehensive research proposal that outline the objectives of the study, methodologies, and measures to protect rights and welfare of the participants. The Ethical Review Board reviewed the proposal in line with established guidelines, focusing on aspects such as informed consent, confidentiality, and the protection of vulnerable participants. Approval was granted, which confirmed that the study met ethical standards for research involving human subjects.

Informed consent was obtained from all participants before their involvement in the study (See supplementary file). Participants were provided with clear information about the research, including its purpose, procedures, potential risks, and benefits. For those interviews conducted on the phone, consent forms were sent electronically to ensure that the participants could review and acknowledge their participation terms before the interview sessions. Participants were also informed of their right to withdraw at any time without repercussions. To protect the privacy of the participants, all collected data were anonymized and securely stored, with access limited to the research team. Sensitive socioeconomic and health-related data were carefully managed to prevent any risk of disclosure. Special care was taken to handle sensitive topics with empathy, particularly when engaging individuals from marginalized communities in the fisheries sector. The research team members were trained to address these issues respectfully, ensuring ethical standards were upheld throughout the process.

## 3 Results

### 3.1 Demographic and socioeconomic information of the FOS and BAU groups

Table 1 depicts the demographic and socioeconomic data of fishers from the sustainability certified and non-certified fishing industries. Most of the respondents in both groups were older than 40 years, with 36.4 % of the FOS fishers between 41 and 50 years, contrary to 30.3 % of the BAU fishers. However, almost equal proportions (approximately 42 %) of respondents from both FOS and BAU were older than 50 years. The majority (> 90 %) of the fishers in both groups were males and both groups comprised married fishers. In terms of employment status, 84.3 % of the FOS-certified workers were permanent staff, while only 15.7 % were temporary staff. For the BAU, 54.6 % were only permanent staff, while over 45 % were temporary staff. Based on their income level, the majority of both groups (FOS = 83.3 % and BAU = 63.3 %), earned between #18,000 and #50, 000 naira per month.

**Table 1:** Demographic and socioeconomic characteristics of the fishers.

Parameters	FOS (*n* = 198)	BAU (*n* = 218)	Total (*n* = 416)
	Freq. (%)	Freq. (%)	Freq. (%)
Age of Fishers (years)			
18–30	3 (1.5 %)	16 (7.3 %)	19 (4.6 %)
31–40	39 (19.7 %)	44 (20.2 %)	83 (20.0 %)
41–50	72 (36.4 %)	66 (30.3 %)	138 (33.2 %)
> 50	84 (42.4 %)	92 (42.2 %)	176 (42.3 %)

Gender			
Male	194 (98.0 %)	206 (94.5 %)	400 (96.2 %)
Female	4 (2.0 %)	12 (5.5 %)	16 (3.8 %)

Marital status			
Single	16 (8.1 %)	16 (7.3 %)	32 (7.7 %)
Married	125 (63.1 %)	188 (86.2 %)	313 (75.2 %)
Married but separated	39 (19.7 %)	0 (0.0 %)	39 (9.4 %)
Divorced	18 (9.1 %)	14 (6.4 %)	32 (7.7 %)

Employment status			
Permanent Staff	167 (84.3 %)	119 (54.6 %)	286 (68.8 %)
Temporary Staff	31 (15.7 %)	99 (45.4 %)	130 (31.3 %)

Income Status			
< 18,000	5 (2.5 %)	11 (5.0 %)	16 (3.8 %)
18, 000–50, 000	165 (83.3 %)	138 (63.3 %)	303 (72.8 %)
51, 000 and above	28 (14.1 %)	69 (31.7 %)	97 (23.3 %)

**Fig. 3:**
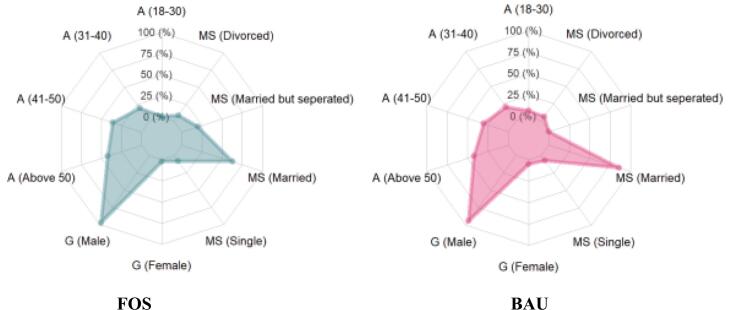
Radar plot of demographic variables between FOS and BAU. A = Age, G = Gender, MS = Marital Status.

The Fig. 3 below shows a graphical view of the FOS and BAU fishing groups. The radar plot compares demographic variables of the FOS and BAU groups. The radar plot shows that the FOS group had fewer married fishers than did the BAU group. Additionally, there were older and more experienced fishers in the FOS group. The groups both had the same proportion of males. The plot shows that FOS certification influenced the workforce demographics, leading to a higher prevalence of older, more experienced males and a lower likelihood of marriage.

### 3.2 Logistic relationship between FOS certification and demographic variables of fishers

Table 2 presents the multivariate logistic results between FOS certification and demographics, using the BAU group as a control to understand the impact of changes in the demographics of the fishers on FOS certification. This logistic regression was computed to establish the relationships between variables of age, gender and marital status, and FOS certification. As shown in Table 2 below, there was a significant positive association between FOS certification and respondents aged 31–40 years and 41–50 years. The probability of FOS certification preferring recruiting the age groups between 31 and 40 years and 41–50 years was higher than those in the age groups between 18 and 30 years and 50 years and above, with odd ratios (ORs) and Confidence Interval (CIs) of “OR, 1.47; CI, 0.054 - 0.515” and “OR, 1.55; CI 0.097 – 0.542” respectively. This demonstrates that FOS certification favored the recruitment and participation of more fishers between the ages of 31 and 50 years. Furthermore, the logistic regression indicated a negative (−0.289) significant association between FOS certification and the involvement of female employees in fisheries. In contrast, the probability of female fishers in joining FOS certification based on odd ratio was less than 1 (0.68). This outcome shows that fewer female fishers/employees are likely to join the FOS compared with their male counterparts. There was a positive association between single fishers (0.49) and married but separated fishers (0.54) and FOS certification. Thus, the probabilities of “single fishers” and “married but separated fishers” occurring in FOS certification were 1.86 and 1.99-fold (odd ratios) respectively greater than the married and divorced fishers. Similarly, no significant association was found between FOS certification and marital status, specifically among married and divorced fishers. However, the divorced group demonstrated a higher probability of obtaining certification compared to the married group. Thus, the tendency of divorced and married fishers to be involved in FOS certification was lower than that of single fishers and married but separated fishers.

**Table 2:** Multivariate logistic regression of the relationships between FOS certification and demographics.

Group	β (Coef.)	Std. Err.	*Z*-value	P-value	Odd ratio	[95 %Conf. Interval]	
_Intercept	0.2536593	0.131321	1.93	0.054	1.35	−0.00449	0.511808

Age of fishers							
18–30	0.212345	0.115674	1.96	0.095	1.22	−0.01102	0.446785
31–40	0.284512	0.117182	2.43	0.016*	1.47	0.054158	0.514866
41–50	0.319753	0.113231	2.82	0.005*	1.55	0.097166	0.542339
Above 50	0.192526	0.112723	1.71	0.088	1.13	−0.02906	0.414114

Gender							
Male	1	1	1	1	1	1	1
Female	−0.289391	0.120090	−2.41	0.016*	0.68	−0.52546	−0.05332

Marital status							
Single	0.492564	0.127567	4.05	0.009*	1.86	0.259168	0.702589
Married	−0.095634	0.093770	−1.02	0.308	0.76	−0.27997	0.088699
Married but separated	0.5425577	0.121696	4.46	0.000*	1.99	0.303331	0.781785
Divorced	0.1330199	0.126542	1.05	0.294	1.07	−0.11573	0.381774
Var (e.Group)	0.2092375	0.014508				0.182576	0.239792

A *p* value is significant at the 5 % level.

### 3.3 FOS sustainability certification as an indicator of health status of the fishers

Table 3 illustrates the relationship between FOS certification and health related dimensions of the fishers. Regarding the entitlement to sick and annual leave, the fishers from the FOS group (100 %) confirmed access to sick leave and annual leave compared with the BAU group (71.1 %). There is a significant (*p* < 0.001) relationship between the entitlement of fishers to sick leave and FOS certification. In addition, the effect size (Phi =0.403) showed that the strength of the relationship between the groups was moderate (Table 3). This suggests the impact of the sick leave of fishers on FOS certification and could further be confirmed by the statement of one of the key informants:

“*Crew members are usually encouraged to go on annual leave, but because of the peculiarity of the industry that they may come back, and their position is already occupied, they usually don't. However, I have been proving to some crew members that they have gone on leave and resumed back to their job when vessels are available. Therefore, they are encouraged to, even when the captain or crew members don't want to because of what they stand to benefit during their voyages, but often they are encouraged to go on leave”.*

**Table 3:** FOS certification and health status of the fishers.

Health indicators	FOS (*n* = 198)	BAU (n = 218)	Total (*n* = 416)	χ^2^ (d.f)	Six.	Effect size
	Freq. (%)	Freq. (%)	Freq. (%)		(2-sided)	
Sick and annual leave						
Access to sick and annual leaves	198 (100.0)	155 (71.1)	353 (84.9)	67.432 (1)	p < 0.001	0.403
No Access to sick and annual leave	0 (0.0)	63 (28.9)	63 (15.1)			

Inability to Work due to Health Issues						
Health issues affected by work	8 (4.0)	63 (28.9)	71 (17.1)	45.300 (1)	p < 0.001	−0.330
No health issues affected by work	190 (96.0)	155 (71.1)	345 (82.9)			

Type of health issues						
Malaria/typhoid	6 (3.0)	59 (28.0)	65 (15.63)			
Fever/cold	2 (1.0)	0 (0.0)	2 (0.48)			
Seasickness	0 (0.0)	2 (0.9)	2 (0.48)			

Health Insurance						
Insured	192 (97.0)	67 (30.7)	259 (62.3)	193.732 (1)	p < 0.001	0.682
Not insured	6 (3.0)	151 (69.3)	157 (37.7)			

Confirmation of health fitness						
Confirmed	185 (93.4)	177 (81.2)	362 (87.0)	13.766 (1)	p < 0.001	0.182
Not confirmed	13 (6.6)	41 (18.8)	54 (13.0)			

Frequency: number of subjects, χ^2^: chi-square value, d.f: degrees of freedom.

Effect size: phi (Φ) or Cramer's V; Sig. (2-sided): probability value (*p* < 0.05).

Regarding the inability of the fishers to work due to health-related issues, it was found that only 4.0 % of the FOS-certified fishers are affected while 17.1 % of the BAU fishers are affected. A follow-up information based on the type of health issues that have been experienced by fishers was also presented. The percentage of respondents in the BAU group (28.0 %) affected by malaria/typhoid was higher than that in the FOS group (3.0 %). FOS fishers (1.0 %) were affected by fever/cold, while the BAU group had no instances (0.0 %). Among the fishers in the FOS group, none (0.0 %) were affected by seasickness, while the BAU group (0.9 %) was also generally unaffected by this condition. There was a significant (*p* < 0.001) relationship between FOS certification and the inability of fishers to work due to health issues. In a further statistical test, the effect size (Phi = −0.330) shows that the strength of the relationship between the groups is significant. Therefore, there is a strong relationship between FOS certification and the type of health issue and condition. This is evident in the direct communication with one of the key informants who said;

*“There had been issues, and there had been occasions where the crew members were unable to work because of issues arising from being at sea. Perhaps they had a major accident or one that could not be treated at sea. Therefore, they have left ashore, and I think given the period, their jobs are retained although most of them are on contracts. Therefore, they are hindered when they have a major injury or when they have a major accident. For some companies that I will not mention, I have witnessed approximately 5; mainly, I think about two chief engineers, one captain, a crew member and one of the shrimp masters”*.

The majority (97.0 %) of respondents from the FOS-certified fisheries group had health insurance compared with the BAU group (30.7 %). There was a significant (*p* < 0.001) relationship between the health insurance of fishers and FOS certification. On a further statistical test, the effect size (Phi = 0.682) shows that the strength of the relationship between the groups is large (Table 3). A key informant from the FDF noted the relevance of sustainability certification in improving social welfare standards within the fisheries industry;

*“Being certified by the FOS has really pushed us to prioritize the welfare of our workers, and health insurance is a big part of that. Right now, our fishers are covered by health insurance. This wasn't always the case. Before certification, we didn't give much thought to worker welfare beyond basic safety protocols. But the FOS certification process requires compliance with certain international labor standards, and that included ensuring access to health care for our workforce”.*

The key informants revealed that they were unaware of the strict regulations on social health insurance for fishers, but oil accrual coverage was confirmed. One of the them mentioned;

*“I wasn't fully aware of the strict regulations around social health insurance for fishers. It's not something that has been emphasized or clearly communicated to us. Our focus has mostly been on operational compliance, like ensuring vessels meet safety standards and adhering to environmental guidelines. The specifics of health insurance as a requirement kind of flew under the radar but I can confirm that oil accrual coverage is in place for our workers. This is to ensures that in situations where accident occur at work, there is at least some financial support for medical expenses or compensation”.*

More (93.4 %) respondents from the group confirmed that health fitness was checked before fishing compared to those in the BAU group (81.2 %). Additionally, there was a significant (*p* < 0.001) association between the confirmation of the health fitness of fishers before fishing and FOS certification. A further analysis of the effect size (Phi = 0.182) revealed that the strength of the relationship between the groups was low. This signifies a weak relationship between FOS certification and the confirmation of health fitness before fishing. According to the FOS Audit report of 2016:

“*Medical service in the company in which there is one doctor and three nurses, a food handler medical screening test is done twice a year by Lagos clinic we check for (VALENTINE NAMANI and FOWLER BASHIRU) last test was done on 05/02/2018 and the doctor certify that there are medically fit to handle food and allied products Annexe 9: Health Care Certificate”.*

(FOS report)

**Fig. 4:**
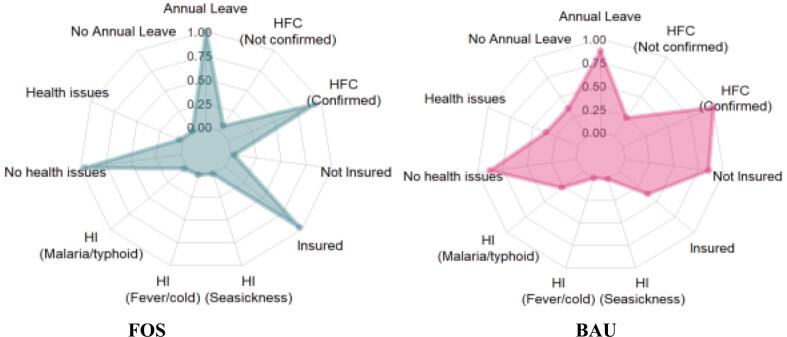
Radar plot of health and safety dimensions between FOS and BAU. HI = health issues, HFC = health fitness confirmation.

The radar plot compares the health variables of fishers from the FOS and BAU groups (Fig. 4). The outcome demonstrated that most FOS-certified fishers are not affected by health issues from work and have health insurance with annual leave, and their health status is confirmed before work.

### 3.4 Logistic relationship between FOS certification and health variables

Multivariate logistic regression was used to determine the relationships between FOS certifications and health variables, including accessibility to sick and annual leave, work-related health issues, type of health issues raised, health issues affecting fishers, health insurance and confirmation of health fitness prior to work (Table 4). There was a negative association between FOS certification and the non-accessibility of fishers to sick and annual leave. The chances of fishers not having access to sick and annual leave based on odds ratio were 0.71 lower than those of fishers having access to sick and annual leave. This suggests a positive impact of FOS certification on fisher's access to sick and annual leave. Similarly, there was a positive (0.07) significant association between FOS certification and not suffering health crises from work, with a higher odds ratio of 1.07. This denotes a lower likelihood of developing work-related health issues among FOS fishers.

**Table 4:** Multivariate logistic regression of the relationship between FOS certification and health.

Group	β (Coef.)	Std. Err	*Z*-value	P-value	Odd ratio	[95 % Conf. Interval]	
Accessibility							
Access to sick and Annual leave	1	1	1	1	1	1	1
No access to sick or annual leave	−0.17401	0.051273	−3.39	0.001*	0.71	−0.2748	−0.07322

Health issues from work							
Affected by health issues	1	1	1	1	1	1	1
Not affected by health issues	0.073579	0.089262	0.82	0.41	1.07	−0.10189	0.249049

Types of health issues							
Malaria/typhoid	−0.32173	0.095941	−3.35	0.001*	0.58	−0.51033	−0.13313
Fever/cold	−0.66284	0.230865	−2.87	0.004*	0.41	−1.11667	−0.20901
Seasickness	0.217785	0.231904	0.94	0.348	1.23	−0.23809	0.673657

Health insurance							
Insured	1	1	1	1	1	1	1
Not insured	−0.66284	0.038595	−17.1	0.00*	0.45	−0.73871	−0.58697

Fitness to work before work							
Confirmed	1	1	1	1	1	1	1
Not confirmed	−0.238756	0.045453	5.25	0.00*	0.67	−0.449406	−0.232806

The p value is significant at the 5 % level.

Further analysis based on specific previous health issues revealed a negative correlation between FOS certification fishers and susceptibility to malaria/Typhoid and fever/cold with values of −0.322 and − 0.663, respectively. The odds ratios of 0.58 and 0.41 imply fewer chances of being affected by malaria/typhoid, and fever/cold, respectively, compared with positive correlation (0.218) obtained for a higher likelihood (odd ratio of 1.27) of developing seasickness. Similarly, there was a significant negative association between FOS certification and the health insurance of fishers. The number of fishers who participated in FOS certification programs and did not have health insurance was 0.67 times lower than the numbers of those who were insured and the odds ratio was also less than 1 (0.45). This indicates that with FOS certification, health insurance is guaranteed. There was also a positive relationship between FOS certification and health fitness at work.

## 4 Discussion

Demographic data are crucial for understanding systemic exploitation and pressure within the fishery, as socioeconomic factors play a significant role in how fishers exploit the common pool. In addition to the dynamics observed in shrimp ecological systems, changes also occur in socioeconomic and ecological systems through demographic transitions [93,94]. The demographic results revealed that most fishers in both groups are aged over 40 years, with FOS-certified company favoring individuals in the 41–50 age range. This finding aligns with the assertion of Criscuolo et al. [95] that middle-aged workers are often prioritized in industries that is physically demanding and requires skill-intensive individuals, such as fishing. Younger fishers (aged 18–30 years) may be underrepresented due to limited experience, while older fishers (above 50 years) may face declining physical fitness, impacting their productivity and safety [96]. The high representation of male fishers (>90 %) in both groups further reflects the gendered nature of the fishing industry, a trend commonly reported in developing countries such as Nigeria. Fishing operations either aquaculture or capture fisheries is traditionally perceived as a male-dominated profession, attributed to cultural norms and the physical demands of the occupation [97]. Women are more likely to be involved in post-harvest activities, such as processing and marketing, rather than active fishing, further contributing to their underrepresentation in demographic data [98].

In terms of employment status, the FOS-certified fishers exhibit a relatively higher proportion of permanent staff compared to the BAU group. This disparity might be due to the influence of certification programs, such that it encourages labor stability. Most of the voluntary sustainability certification standards often encourage better labor practices, including formalized employment and contractual stability, as part of their compliance requirements [99]. Conversely, the higher proportion of temporary workers observed in the BAU group may indicate a lack of structured labor policies, which tends to increase worker vulnerability and reduces access to benefits such as insurance and paid leave. Furthermore, the income levels also differ between the two groups, with a greater proportion of FOS fishers earning more than the BAU workers. Brown & Getz [100] affirmed that certification programs, such as the FOS initiative, often promote improved wages and working conditions by encouraging fair pricing and market access. The higher income levels of the certified fishers may indicate economic advantages of certification, such as premium pricing for certified products and more negotiating leverage for employees.

Despite the fact that fishing is widely recognized as one of the most hazardous working environments, characterized by external risks such as adverse weather conditions, internal organizational pressures, socioeconomic constraints, and other underlying factors often compel fishers to persevere in their operations. The findings in Fig. 4, Table 3, Table 4 on access to sick leave are contrary to the report of ILO [101] on the research of fishing workers in Thailand, such that approximately 50 % of respondents revealed that they do not have sick leave, and many other fishers (approximately 60 %) confirmed that they have no off days a week. Additionally, 30 % of the women were entitled to sick leave. Having access to sick and annual leave is determined by fishing income, such that employees with low wages may not have a secure job, thereby finding it challenging to receive sick leave and payment during periods of illness. Fishing companies in Morocco pay their workers well and offer most workers work-related medical benefits, including paid sick leave [102]. According to the study of ILO, in the case of the Norwegian commercial fishing fleet, the sick leave rate among fishers is notably lower than that observed in other professions [5]. This could be attributed to the physical nature of fishing, whereby individuals with high level of resilience and physical fitness are mostly employed, which may contribute to lower health issues that demand medical leave. In addition, most industrial fishers are employed to work in profit sharing systems, which tends to trigger economic pressures that require minimal absences. For examples, taking sick leave may directly affect their income or portion of the catch, which is different from the salary employees in other professions who typically receive stable wages during illness. Nonetheless, all these factors may lead to significant underlying health issues, as fishers often postpone seeking medical attention due to the demands of their occupation until their condition becomes severe. This assertion could be supported by findings of Lopez-Ercilla et al. [103], who observed that small scale fishers spend more time in hospitals than other working groups in the Mexican community. Fishing is one of the most hazardous jobs, and fishers are primarily absent from work due to muscular or skeletal issues and work-related injuries [5]. Furthermore, the increased access to sick leave and annual leave observed in the FOS-certified group could be due to the substantial economic profit available to their companies once certified.

The findings in Fig. 4, Table 3, Table 4 on the inability to work due to health issues indicate that most workers are not affected by health issues at the workplace. According to the work of ILO [101], approximately 10 % of respondents who engaged in Thai fishing noted that their work is hazardous, with a risk of injury or harm, while most fishers reported sickness due to fishing operations and working conditions. However, they highlight the need for better safety practices and more training for workers to reduce injury or illness. The results of this study diverge from those of Fiorella et al. [104] who investigated the impact of human health on fishing practices in Lake Victoria, Kenya and established that poor health conditions affect fishing activities. Almost all the FOS-certified fishers have not been victims of health issues that could have stopped them from fishing. This result outcome could be attributed to several factors, including the implementation of well-structured health and safety policies, regular training programs, and adherence to industrial certification standards. Our study also found that workers from the FOS-certified company have access to proper medical facilities and protective equipment, which could have contributed to the lower health issues among the fishers. As illustrated by a key informant, the FDF usually assesses the health conditions of fishers before embarking on fishing operations, with a standby nurse for inspection.

Our results on the type of health issues affecting the fishers are similar to the findings of Kébé [105] on the livelihood analysis of Liberia's coastal fisheries. It was reported that the most prevalent diseases affecting the fishing community were malaria, diarrhea, and pneumonia, which have unfavorable impacts on the health of the fishing community. Matheson et al. [106] reported significant health implications for fishers in the industrial fishing sector, noting that the hospital admission rate among fishers was approximately 60 %. Most harvesters reported were diagnosed with illnesses such as digestive, cardiovascular, mental, urinogenital, respiratory, and nervous conditions. Correspondingly, in the British case study of trawl fishers, many harvesters were ill due to gastrointestinal, respiratory, and dermatological illnesses, although a few had depression. In contrast, findings from our study show a considerably lower prevalence of health issues, with only 4 % and 28.9 % of fishers in FOS-certified and BAU groups respectively, reporting health-related concerns. This disparity highlights potential differences in health management practices, workplace conditions, or access to healthcare. Spanish fishers were also diagnosed with digestive and eye problems [107]. Similarly, among Polish fishers, circulatory, nervous, and genital diseases are common causes of observed disease [108]. The findings of this study are also similar to the work of Grimsmo-Powney et al. [109] on the occupational health requirements of commercial fishers in southwestern England. Based on emergency evaluations, commercial fishers have required medical attention after returning from fishing because of major injuries such as fractures, amputations, cardiac arrest, appendicitis, renal colic, and asthma. The most common illnesses affecting fishers are infectious or muscular, and skin diseases are sometimes caused by allergies. In the context of Nigerian fisheries, FOS certification has not yet significantly alleviated health issues among fishers, as the majority of fishing industries have yet to adopt or subscribe to this sustainability program. However, the only FOS-certified company in Nigeria, ASL, has engaged health specialists to monitor the health status of its fishers. This practice can be attributed to the influence of the FOS program, as employing health specialists is one of the mandatory requirements considered for certification under this sustainability standard. It is deemed essential for all fishing vessels to have access to a standby nurse in case fishers face health issues while on board.

The findings in Fig. 4, Table 3, Table 4 on health insurance shows that most of the FOS-certified fishers are insured. This outcome is contrary to the finding of Kaustell et al. [110] on the predictors of occupational injuries in Finnish commercial fisheries. Health insurance was made compulsory for fishers with adequate income. Approximately 13 % of the respondents joined the Occupational Health Insurance Scheme (OHIS), and a few fish were given insurance checks. Favourably, Barr et al. [111] confirmed the importance of a premium to support the health insurance of fishers taking part in certification programmes. The fact that many FOS-certified fishers are insured suggests that the certified company places greater importance on insurance compared to non-certified companies, as insurance is advantageous within the context of the FOS certification program. This observation aligns with our findings, which reveal that most non-certified BAU fishers lack insurance coverage. Furthermore, the price premium is also considered a factor, with the FOS certification covering the cost of health insurance for participating fishers. The cost of health insurance has well been documented as a factor that influence health insurance uptake [112]. High insurance costs, even with tiered subsidies based on income remain a barrier to comprehensive coverage in fisheries [113]. Health insurance costs appear to play a significant role in shaping access to coverage among fishers. In our study, the key informants revealed limited awareness of regulations surrounding social health insurance for fishers, but it was confirmed that oil accrual coverage is available to provide financial support for medical expenses or compensation in case of workplace accidents. Nevertheless, comprehensive health insurance, which encompasses a wider range of medical requirements, is uncommon, probably due to the related expenses. For fishing industries, the availability of employer subsidized healthcare significantly affects the affordability and accessibility of insurance. Our study aligns with the findings of Lewis-Smith et al. [114], which demonstrate a positive correlation between risk and insurance coverage for fishers in the commercial fishing fleets, United States. Fishing industries as well as the fishers may assess the probability of nonfatal injuries in their operations when deciding on health insurance coverage. The inherent risks of injury can vary across fisheries, influencing both the perceived value of insurance and the willingness to pay for it [114]. This variation in risk management practices likely explains the disparity in insurance uptake between FOS-certified fisheries, where 97 % of workers are insured, and BAU fisheries, where only 30 % have insurance coverage. However, unlike the Affordable Care Act compliant insurance models in the United States, where premiums are not based on individual health risks [114], the Nigerian fishing companies lacks uniformity in coverage access.

The outcomes in Fig. 4, Table 3, Table 4 confirm that the health status of the fishers was confirmed before they worked. According to the interview responses, medical fitness was always considered before fishing operations. Each time the crew is ready for boarding, there are rigorous medical checks and tests on the fishers. If a fisher is found suitable for work, the nurses intervene, officially deeming their condition unfit for work. It was reported that in the Republic of Korea, workers under the age of 18 should conditionally produce medical confirmation for fitness before working [115]. According to the ILO, Nigeria is among the countries that adopt initial medical examinations at intervals to confirm whether workers are fit to work and to avoid contagious diseases among workers. This experience is contrary to the research of Grimsmo-Powney et al. [109] on the occupational health requirements of fishers in southwestern England. Some respondents reported returning to shore in emergencies due to medical issues requiring consultation with their doctors. Consequently, they were unable to continue working during these periods. In contrast, our study congirmed that some workers had no access to dental services. Our study revealed that the fishers from the FOS-certified ASL fishing industry appear more organized and healthier, which is explainable given the health benefits offered to them. This situation is related to their obligations to government regulations and financial situations, as they hold the largest share of the Nigerian fisheries industry, with a great deal of financial commitment. The FOS certification programme regulates access to sick and annual leave, and insurance is government-mandated in certain situations. Annual routine medical checks are undertaken by medical personnel, who check their fitness to sail through preventive measures.

### 4.1 Conclusions

This research examines the health-related dimensions of fishers and the impact of fishing operations within Nigerian shrimp fisheries in the Atlantic Ocean, particularly in the context of sustainable certification programs. The objectives were achieved by analyzing the relationships between various health-related factors, demographic characteristics, and the FOS sustainable certification program, while also comparing the outcomes with those of fishers in non-certified BAU fishing industries. The well-being of fishers is a critical component of both human health and the broader socio-ecological system. Sustainable seafood certification programs, such as the FOS initiative, must therefore actively incorporate fisher health into their frameworks. This study confirmed that the FOS-certified fishing industry takes into account key demographic and socioeconomic variables, such as age, marital status, gender, and employment type, during its selection process. The health and safety provisions of the FOS sustainability certification initiative include health insurance, verification of health fitness before work, causalities from work, accessibility to sick and yearly leave, and incapacity to work owing to health difficulties. Comparatively, the fishing industry participating in sustainability certification programs demonstrates greater efforts to improve the health and safety of their workers than their business-as-usual (BAU) counterparts. These findings are significant given the intertwined nature of SDG 3, which focuses on health and well-being, and SDG 14, which emphasizes life below water. This suggests that fisher health is critical not only for the welfare of individuals but also for maintaining socio-ecological dimension of sustainable fishing practices and healthy marine ecosystems. Overall, the study emphasizes that fishers, who are key actors in the socio-ecological system, are directly influenced by their working environment, including exposure to marine ecosystems, environmental changes, and occupational hazards. Hence, certification programs like FOS that integrate health dimensions into their sustainability criteria not only promote individual wellbeing but also contribute to healthier ecosystems by reducing unsustainable practices, promoting ecological resilience, and ensuring that fishers have the capacity to manage their role in maintaining sustainable fisheries.

### 4.2 Recommendations for improving the health of fishers and safety practices

The study highlights the risks associated with industrial fishing operations as well as the intricate relationship between workplace conditions, access to healthcare, health insurance coverage, and health outcomes for fishers. Based on the findings of the study, it is important to propose novel strategies and challenges to enhance health and safety practices. First, evidence from this study indicates that FOS-certified fishers benefit from regular medical assessments, which contribute to their reduced incidence of health issues. Therefore, all fishing companies should be mandated to adopt sustainability certification standards that conduct health screenings to assess crew fitness before departure. This approach can prevent severe health issues from arising during fishing operations. In addition, routine medical checks and implementation of preventive healthcare measures should be ensured to address the health risks in the fishing industry. The presence of trained medical personnel on industrial fishing vessels should be made mandatory, following the example of FOS-certified operations. Furthermore, fishing companies should collaborate with healthcare providers to execute preventive health initiatives, provide vaccinations, dental services, and treatments for prevalent ailments such as malaria, pneumonia, and gastrointestinal infections.

Second, our findings demonstrate the need to enhance awareness and compliance with health insurance regulations within the fisheries industries. Limited knowledge of social health insurance among key informants implies the need for all the fishing companies to conduct regular educational workshops that focus on insurance policies and healthcare rights. These workshops should be incorporated into existing training programs to ensure comprehensive understanding among workers. Furthermore, collaboration between fisheries policymakers and the fishing companies is essential to simplify and vdisseminate insurance guidelines. Subsidized insurance schemes, supported by government programs and certification initiatives like FOS, should be expanded to reduce the cost barrier for low-income fishers.

Third, access to paid sick leave and work-related benefits should be improved to bridge the gap observed between FOS-certified and BAU fishers. Thus, minimum standards for paid sick leave across the fishing sector should be established to ensure that workers can take time off without income loss. Government labor agencies must introduce and enforce these standards to protect vulnerable workers. Certification programs, such as FOS, should make paid sick leave and work-related medical benefits a mandatory requirement for certification, incentivizing companies to prioritize worker welfare. Furthermore, companies engaged in profit-sharing arrangements ought to implement income support policies to alleviate the economic burdens that discourage employees from utilizing essential sick leave. It is also essential to strengthen the safety practices through training, and the enforcement of standards is crucial to mitigating the risks inherent in fishing. Comprehensive safety training should be mandatory for all fishers to gain proper knowledge about equipment handling, first aid, and emergency response procedures. The study highlights the need for greater enforcement of protective gear usage, such as gloves, helmets, and harnesses. Governments and certification initiatives must mandate the use of such equipment and provide financial assistance to companies struggling with associated costs. Increased regulatory inspections of fishing vessels are also necessary to ensure compliance with safety standards, with penalties for companies failing to meet these requirements.

Finally, financial support or grants from governments and NGOs must reduce economic disparities between fishing companies, enabling smaller companies to improve their health and safety measures. Policy incentives such as lowered taxes or subsidies should be encouraged to boost enrollment in sustainability certification programs. Additionally, the establishment of robust health data collection systems would enable fishing companies to track the health outcomes of fishers and implement evidence-based interventions. Enhancing certification coverage by providing financial and logistical assistance to organizations throughout the certification process would augment the beneficial effects noted in FOS-certified fishing company.

## Author statement

The authors affirm that this study, titled *“Health-Related Dimensions of Fishers for Sustainable Commercial Fisheries in the Atlantic Gulf of Guinea: Ecological and Social Assessments,”* is an original contribution to the understanding of the intersection between sustainable fisheries certification programs and the health and well-being of fishers in the Gulf of Guinea region.

**Responsibilities**

The authors were collectively responsible for the following:

1. **Study Design and Conceptualization:**

The conceptual framework was developed to explore the health-related dimensions of fishers, integrating both ecological and social assessments.

2. **Data Collection and Analysis:**

Quantitative and qualitative data were collected and analyzed, focusing on demographic, socioeconomic, and health-related variables to compare sustainability-certified fisheries with non-certified fisheries.

3. **Ethical Considerations:**

Informed consent was obtained from all participants before their inclusion in the study. The study adhered to strict ethical guidelines to ensure data anonymity, sensitivity, and integrity.

4. **Manuscript Preparation:**

The manuscript was collaboratively drafted, reviewed, and revised to ensure its accuracy and relevance to the broader field of sustainable fisheries and fishers' health.

5. **Funding and Support:**

The study was conducted without any external influence from funding bodies or commercial interests that could have impacted the findings or conclusions.

## Funding

This research was not funded by any organization.

## CRediT authorship contribution statement

**Isa Olalekan Elegbede:** Writing – review & editing, Writing – original draft, Visualization, Validation, Supervision, Methodology, Investigation, Funding acquisition, Formal analysis, Data curation, Conceptualization. **Saud M. Al-Jufaili:** Writing – review & editing, Visualization, Supervision, Methodology, Data curation. **Toheeb Lekan Jolaosho:** Writing – review & editing, Writing – original draft, Visualization, Validation, Methodology, Investigation, Formal analysis, Data curation. **Babalola Tesleem:** Writing – review & editing, Writing – original draft, Validation, Methodology, Data curation, Conceptualization. **Awe Folalu Adekunle:** Writing – review & editing, Visualization, Methodology, Investigation, Data curation. **Olarinmoye Oluwatosin Modupe:** Writing – review & editing, Visualization, Validation, Supervision, Methodology, Formal analysis, Conceptualization. **Salisu Monsuru Adekunle:** Writing – review & editing, Visualization, Supervision, Methodology, Funding acquisition, Data curation. **Adedeji-Adenola Halimat:** Writing – review & editing, Writing – original draft, Validation, Investigation, Formal analysis.

## Declaration of competing interest

The authors declare no conflicts of interest in relation to this study.

## Data Availability

Data will be made available on request.

## Appendix A Supplementary data

Supplementary material: Part A: Informed consent for the Participants; Part B: Survey questions for the Industrial fishers and the Key informants. Image 1
