# Supplementary material for: Health-related dimensions of fishers for sustainable commercial fisheries in the Atlantic Gulf of Guinea: Ecological and social assessments
Source: One Health. 2025 Jan 10;20:100972. doi: 10.1016/j.onehlt.2025.100972 (PMC11791358; doi:10.1016/j.onehlt.2025.100972)
Supplement: Supplementary file 1 — Supplementary material: Part A: Informed consent for the Participants; Part B: Survey questions for the Industrial fishers and the Key informants. [file mmc1.docx]

**SUPPLEMENTARY FILE**

**Informed Consent Form**

**Title of Research:**

Health-Related Dimensions of Fishers for Sustainable Commercial Fisheries in the Atlantic Gulf of Guinea: Ecological and Social Assessments

**Research Team:**

Elegbede Isa Olalekan, Al-Jufaili Saud M, Jolaosho Toheeb Lekan, Babalola Tesleem, Awe Folalu Adekunle, Olarinmoye Oluwatosin Modupe, Salisu Monsuru Adekunle, Halimat Adedeji-Adenola

**Introduction:**

You are being invited to participate in a research study conducted by the research team. The purpose of this study is to assess the health-related dimensions of fishers in commercial fisheries to promote sustainable practices in the Atlantic Gulf of Guinea. Your participation is entirely voluntary, and you may withdraw at any time without any consequences.

**Purpose of the Study**

This study aims to explore the ecological and social factors affecting the health and well-being of fishers in commercial fisheries. The results will help develop strategies for sustainable fisheries that prioritize both environmental and human well-being.

**What Participation Involves**

- **Procedures:**
  If you agree to participate, you will be asked to provide information during an interview. This may be conducted in person or via phone. The interview will include questions about your health, work conditions, and socioeconomic status.
- **Duration:**
  Each interview will last approximately 30-60 minutes

**Potential Risks and Benefits**

- **Risks:**
  While there are no significant risks anticipated; some questions may touch on sensitive personal topics. You may choose not to answer any question you find uncomfortable.
- **Benefits:**
  The study findings will contribute to policies aimed at improving the health and safety of fishers, potentially benefiting fishing communities in the long term.

**Confidentiality and Data Protection**

Your privacy is our priority. We guarantee that all the information you provide will be:

- Anonymized to ensure your identity is not disclosed.
- Stored securely, with access restricted to the research team.
- Used solely for the purposes of this research.

**Voluntary Participation and Right to Withdraw**

Your participation is completely voluntary. You have the right to:

- Decline to answer any question.
- Withdraw from the study at any time, without providing a reason or facing any repercussions.

**Contact Information**

If you have any questions or concerns about this study, you can contact on our Email, and Phone Number.

**Consent Declaration**

I have read and understood the information provided above. I understand the purpose, procedures, risks, and benefits of the study. I acknowledge that my participation is voluntary, and I can withdraw at any time without consequences.

I consent to participate in this study:

- ☐ Yes ☐ No

I consent to the use of anonymized data for research purposes

☐ Yes ☐ No

I consent to the interview being recorded for accuracy

☐ Yes ☐ No

**Participant's Name:**

_________________________________
**Participant's Signature:**

_____________________________
**Date:**

_____________________________________________

For electronic consent (if interview is conducted remotely):

☐ By checking this box, I confirm that I have read and understood the informed consent form and agree to participate in the study.

**Researcher’s Name:**

_________________________________
**Researcher’s Signature:**

_____________________________
**Date:**

_____________________________________________

Research Questionnaire

Health-Related Dimensions of Fishers for Sustainable Commercial Fisheries in the Atlantic Gulf of Guinea: Ecological and Social Assessments

Section A: Demographic and Socioeconomic Characteristics

Please fill out the following details by choosing one of the options provided:

1. Age Group:

☐ 18–30

☐ 31–40

☐ 41–50

☐ > 50

2. Gender:

☐ Male

☐ Female

3. Marital Status:

☐ Single

☐ Married

☐ Married but separated

☐ Divorced

4. Employment Status:

☐ Permanent Staff

☐ Temporary Staff

5. Income Level in Naira:

☐ < 18,000

☐ 18,000–50,000

☐ > 51,000

**Section B**: Health Status Indicators

6. Do you have access to sick and annual leave?

☐ Yes

☐ No

7. Has your health ever been affected by work?

☐ Yes

☐ No

8. If yes, what type of health issues have you experienced due to work? (Select all that apply):

☐ Malaria/Typhoid

☐ Fever/Cold

☐ Seasickness

☐ Other (please specify): ________________________

9. Do you have health insurance?

☐ Yes

☐ No

10. Was your health fitness confirmed before employment?

☐ Yes

☐ No

**Section C**: Semi-Structured Open-Ended Questions for Key Informants

11. Health Insurance provision

Are fishers typically covered by health insurance in this industry?

What are the main challenges to ensuring fishers have access to health insurance?

**12. Health Insurance Regulations and requirements**

How familiar are you with the specific regulations surrounding social health insurance for fishers?

What do you think could be done to improve awareness and understanding of these regulations among employers and fishers?

Have health insurance requirements for fishers been clearly communicated to your organization?

In what ways could these requirements be better explained by regulators or policymakers?

**13. Focus on Operational Compliance**

How do you see health insurance fitting into operational compliance framework (e.g., safety standards and environmental guidelines)?

**14.** **Oil Accrual Coverage**

Can you elaborate on the oil accrual coverage you provide for workers?

How does this coverage compare to the requirements or benefits of social health insurance?

What are the strengths and limitations of this type of coverage in addressing the health and safety needs of fishers?

**15. Support for Medical Expenses and Compensation**

How effectively does the current financial support system handle medical expenses or compensation for accidents?

**16. Leave Policies**

How often are fishers granted sick or annual leave?

What factors determine whether a fisher can take leave?

**17. Workplace Health Challenges**

What are the most common health challenges faced by fishers during operations?

How are health emergencies typically addressed at sea?

**18. Accidents and Injuries**

Can you describe the types of accidents or injuries commonly encountered during fishing operations?

What measures are in place to prevent or manage such incidents?

**19. General Observations**

What recommendations would you make to improve the health and safety of fishers?

Are there any specific policies or practices you think should be implemented?

Instructions for Completion:

This questionnaire is intended to gather information about your experiences and perspectives.

If you feel uncomfortable answering any question, you may choose not to respond.

Your responses will remain confidential and will only be used for research purposes.

Thank you for your participation!
